# Development and validation of a disease-specific quality of life questionnaire for patients with peripheral artery disease (QOLPAD)

**DOI:** 10.1186/s41687-022-00451-0

**Published:** 2022-05-27

**Authors:** Ebru-Berrin Arman, Matthias Augustin, Nicole Mohr, Sebastian E. Debus, Peter Breuer, Christine Blome

**Affiliations:** 1 Institute for Health Services Research in Dermatology and Nursing (IVDP), University Medical Center Hamburg-Eppendorf (UKE), Martinistraße 52, 20246 Hamburg, Germany; 2 Department of Vascular Medicine, University Medical Center Hamburg-Eppendorf (UKE), Hamburg, Germany; 3 Department of Vascular and Endovascular Surgery, Asklepios Klinik Wandsbek, Hamburg, Germany

**Keywords:** Health-related quality of life, Peripheral artery disease, Patient-reported outcomes, Validation, Questionnaire

## Abstract

**Background:**

The aim of this study was to develop and validate a short and feasible questionnaire to measure health-related quality of life (HRQoL) in patients with peripheral artery disease (PAD). The content of the new instrument is intended to correspond with the simultaneously developed instrument Patient Benefit Index for PAD (PBI-PAD), which evaluates treatment goals and benefits in this patient group.

**Methods:**

Fifty patients stated their disease burden on free-text questionnaires, which was used by an interdisciplinary expert panel to develop 12 items for the new instrument, named Quality of Life questionnaire for patients with peripheral artery disease (QOLPAD). The validity of the instrument was tested in patients from Germany with PAD stages I to IV who completed the QOLPAD, EuroQol questionnaire (EQ-5D-3L; EuroQol visual analogue scale (EQ VAS)), and Vascular Quality of Life questionnaire (VascuQoL) before (baseline) and three months after (follow-up) treatment.

**Results:**

One hundred and three patients were included at baseline (mean age: 68.6 years; 68% male), among whom, 57 provided data at follow-up. Most patients (86.4%) rated the completion of QOLPAD as being easy. Internal consistency was satisfactory, with a Cronbach’s alpha of 0.74 (baseline) and 0.84 (follow-up). Convergent validity was indicated by significant correlations with the EQ-5D-3L (baseline: − 0.62; follow-up: − 0.81), EQ VAS (baseline: − 0.44, follow-up: − 0.79), VascuQoL global score (baseline: − 0.77; follow-up: − 0.87), global rating of impairment (baseline: 0.64; follow-up: 0.71), and PAD stage (baseline: 0.40; follow-up: 0.67). Sensitivity to change was confirmed by significant correlations of change in the QOLPAD with changes in convergent criteria; however, the high number of dropouts limits the generalizability of this finding.

**Conclusion:**

This study provided evidence that the QOLPAD is internally consistent and valid in patients receiving treatment for PAD in Germany.

## Introduction

More than 200 million people worldwide have peripheral artery disease (PAD) [1], which predominantly occurs in elderly people, with a prevalence of over 10% at ages 60–70 years [2]. PAD is caused by arteriosclerosis of the lower extremities. Patients with PAD stage I (as classified according to Fontaine [3]) show no symptoms, and PAD usually manifests clinically as a shortened walking distance due to leg pain (intermittent claudication, PAD stage II) [2–4]. PAD stage II is subdivided into stages IIa and IIb depending on a painless walking distance of > 200 m (stage IIa) or < 200 m (stage IIb). In more advanced stages III and IV, patients report rest pain, ulcerations, or gangrene with a risk of limb loss.

Clinical parameters and health-related quality of life (HRQoL) are important outcomes for the evaluation of treatments (e.g., pharmaceuticals, surgery, or lifestyle changes) [5, 6]. Both generic instruments and disease-specific instruments can be used to determine the HRQoL; however, disease-specific instruments are generally more sensitive in detecting changes [7].

The Vascular Quality of Life questionnaire (VascuQoL) is a frequently recommended disease-specific questionnaire for HRQoL in patients with PAD [7–9]. The VascuQoL consists of 25 items assessing the domains of pain, symptoms, and activities, as well as social and emotional impairments. Completion of this questionnaire takes an average of 9.6 min [10]. Recently, a short version of VascuQoL with only six items (VascuQoL-6) has been developed and has shown good psychometric properties in a validation study [11].

A systematic review by Poku et al. (2016) identified six generic and seven disease-specific HRQoL instruments that have been validated in patients with PAD, but no study has provided evidence for a full psychometric evaluation (including internal consistency, test-retest reliability, content and construct validity, responsiveness, floor-ceiling effects, and acceptability) [12]. The evaluation of floor and ceiling effects and acceptability is missing in the VascuQoL (25 item version). In a systematic review, Conijn et al. rated the content validity of VascuQoL as positive but highlighted a lack of information on internal consistency and structural validity [13].

A systematic review by Aber et al. identified that a complete instrument for assessing the quality of life of patients with PAD should include the following six topics: symptoms, impact on physical functioning, impact on social functioning, psychological impact, financial impact, and process of care. However, they also found that none of the existing HRQoL questionnaires covered all six dimensions; in particular, employment-related aspects were not included in any of them [9].

The aim of this study was to develop de novo and validate a simple and short disease-specific instrument that measures the relevant aspects of HRQoL in patients with PAD (Quality of Life questionnaire for patients with peripheral artery disease, QOLPAD) for use in research and clinical practice. Through the development of the questionnaire, we also aimed to create an instrument that corresponds to the Patient Benefit Index for PAD (PBI-PAD), which evaluates the treatment goals and benefits in patients with PAD [14]. Both constructs, HRQoL and patient goals and benefits, are highly relevant for clinical decision-making. For other disease groups, we found that patients’ treatment goals can differ from the extent of impairment in their respective functions and that treatment goals are often still considered important by patients, even if these goals have already been achieved to a high extent [15]. Thus, the instruments aim to assess the same content, but focus on current impairment (QOLPAD) or treatment needs/benefits (PBI-PAD).

The combined use of QOLPAD and PBI-PAD is also recommended because of the impairment by response shift bias and recall bias [16]. While benefit assessment via changes in HRQoL can be impaired by response shift, direct benefit assessment via PBI can be impaired by recall bias. Thus, combined use can enhance confidence in the findings regarding treatment benefits.

## Patients and methods

A prospective, non-interventional study was performed in patients with PAD and consisted of two phases. Participants in both phases were recruited at the Vascular Surgery Department of the University Medical Center Hamburg-Eppendorf (UKE) and the Asklepios Klinik Wandsbek in Hamburg, Germany. The QOLPAD was developed in German.

### Phase 1: instrument development

In the first phase, the new instrument was inductively developed based on open-item collection using free-text responses from patients. To identify patient-relevant aspects of HRQoL impairments due to PAD, 50 adult patients who received inpatient treatment at the UKE and who were fluent in German completed an open questionnaire with questions referring to their PAD-specific health problems and treatment goals. First, patients were asked to describe their PAD-related impairments and needs in general (“Please describe in your own words whether and in which way you are burdened in your life by the circulatory disorder of the legs,” and “Please describe which goals and benefits would be of particular importance for you in the treatment of the circulatory disorder”). Subsequently, open questions were asked for additional impairments and goals related to different areas of life, such as social life. All of the data were transcribed and tabulated, resulting in the following domains: physical impairment, everyday life, working life, spare time/social activities, treatment, and psyche.

Based on these data, an expert panel consisting of one dermatologist, one specialist in vascular and endovascular surgery, one medical student, one expert in healthcare research, and one psychologist specializing in HRQoL measurement and instrument validation determined 12 items for the QOLPAD that covered the results of the open survey. The exact number of items and domains had not been previously defined; instead, both the number and content of the items were based on patient statements. To enable good recollection by the patients, all items in the QOLPAD refer to the seven days prior. A uniform response scale was used to ensure that the instrument was short and simple.

In subsequent cognitive debriefing, the resulting preliminary version of the QOLPAD was tested in seven patients who completed the questionnaire, followed by interviews on the practicability and comprehensiveness of the questionnaire with regard to patient-relevant topics. During these interviews, no missing content relevant to HRQoL was determined; however, the wording of some items or questions was changed.

### Phase 2: validation study

During the second phase of the study, the QOLPAD was psychometrically tested in a longitudinal validation study conducted between February 2014 and October 2015. The QOLPAD was validated in 103 patients treated at two vascular surgery departments in Hamburg, Germany.

The inclusion criteria were as follows:

- ≥ 18 years of age
- medically confirmed PAD (based on blood flow tests using a Doppler probe and/or angiogram) for which treatment was scheduled
- sufficient mental, physical, and linguistic abilities to participate in the questionnaire study
- obtained written informed consent after the patients had been informed about the study by a physician
- treated under the supervision of medical staff or physicians

PAD treatment supervised or conducted by medically trained personnel was chosen as an inclusion criterion to include patients for whom a change in HRQoL could be expected, allowing the determination of whether the QOLPAD was sensitive to change. Consequently, patients who merely recommended unsupervised actions, such as lifestyle changes or exercise, were excluded from the study. As we did not aim to measure the effect of a particular intervention, the treatment was not specified in the questionnaire; thus, a patient sample receives different types of treatment (e.g., supervised exercise, surgery).

Data were collected at two time points: at the first visit (baseline) before the onset of a new treatment, and three months (± 2 weeks) after or during treatment (follow-up). A recall period of three months was selected to better differentiate between disease- and treatment-related complaints. At UKE, all participants were inpatients at baseline. At Asklepios Klinik Wandsbek, outpatients were recruited at consultation hours (baseline), while the second measurement took place either at the hospital (if patients had a follow-up appointment) or by mail.

At both time points, patients self-completed the QOLPAD without help from the study personnel. In addition, patients stated in a questionnaire how easy or difficult it was for them to complete the QOLPAD. They also completed the three-level version of EQ-5D-3L on generic HRQoL, along with the EuroQol visual analogue scale (EQ VAS) on subjective health [17] and the VascuQoL [10]. The EQ-5D-3L and EQ VAS global scores have a possible range from 0 to 1, with higher values indicating better health. The VascuQoL has a range from 1 to 7, with higher values indicating better HRQoL.

Data assessment also included sociodemographic information, including age, sex, education, and current employment (baseline). Furthermore, patients completed several global rating items on a 5-point Likert scale regarding current impairment due to PAD (from “*not at all*” to “*very much*”; baseline and follow-up), evaluation of treatment effectiveness (from “*not effective*” to “*very effective*”; follow-up), recommendation of treatment to other patients (from “*not at all*” to “*definitely*”; follow-up), and symptom improvement (from “*much better*” to “*much worse*”; follow-up). The PAD stage, classified according to Fontaine [3], was determined by a physician at both time points.

If the patient had answered at least 75% of the total items, the QOLPAD global score was calculated as the overall arithmetic mean; otherwise, no global score was computed (no missing data imputation was performed). Reversely formulated items (Items 6 and 10) were recoded. Floor and ceiling effects were determined as the percentage of patients with the highest or lowest possible QOLPAD global scores at the baseline and follow-up, respectively.

Cronbach’s alpha was calculated to measure the internal consistency of the QOLPAD. Convergent validity was determined using the Spearman’s correlation coefficient of the QOLPAD with convergent criteria (generic and disease-specific HRQoL, global ratings as described above, and PAD stage). Sensitivity to change was analyzed via the correlation between the change in QOLPAD from baseline to follow-up and the change in the convergent criteria. For the global ratings that were assessed at follow-up only (recommendation to others, effectiveness, and symptom improvement), their correlation with QOLPAD change was determined. All statistical analyses were performed using IBM SPSS Statistics version 23 (Armonk, NY, USA) for Windows.

**Fig. 1:**
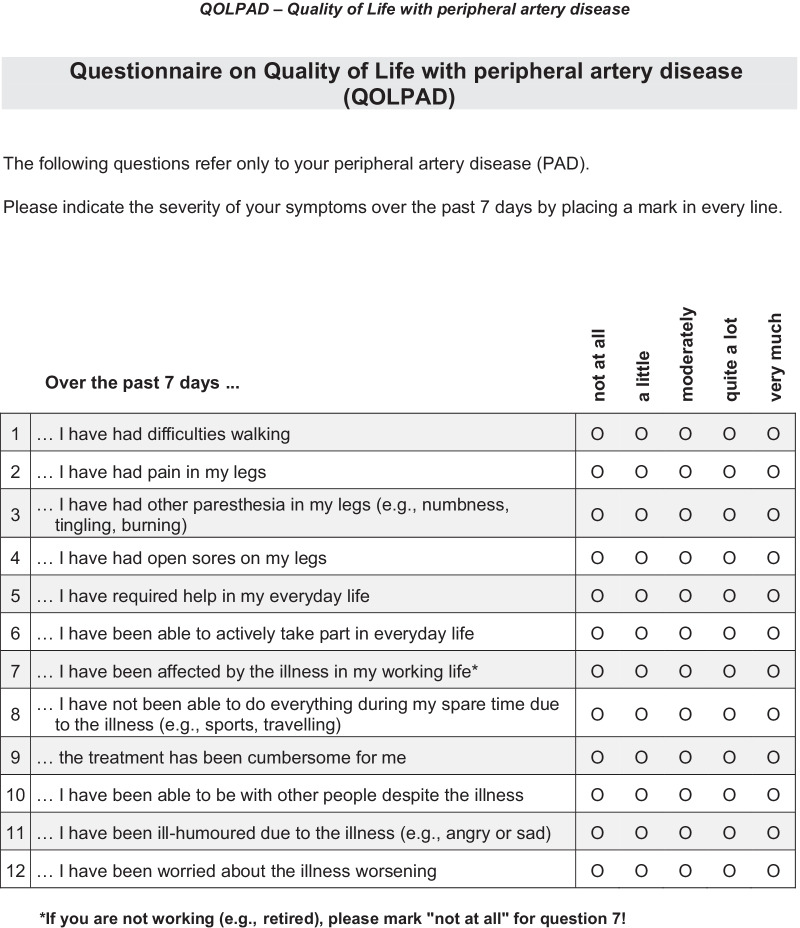
British English version of the QOLPAD

The original German questionnaire was translated into British English for the purpose of this publication (Fig. 1); this involved independent forward translations by two native speakers of the target language (professional translators), independent back-translations by two German native speakers (professional translators), consensus finding with translators, and proofreading by another British English native speaker. The English version has not yet been tested in patients; in this study, only the German version was used.

## Results

### Development of the QOLPAD

In the survey, patients reported 89 different aspects of HRQoL. The answers were sorted according to topic and frequency. The expert panel combined similar related aspects into more general terms, which led to 37 non-redundant items in the first step and 12 final items in the second step. Examples of treatment-related burdens reported by patients were “having to take many pills” (mentioned three times), side effects (mentioned once), frequent visits to the doctor and physiotherapy (mentioned six times). These aspects were first summarized into the categories “medication” and “expenditure of time.” In the phase of further generalization of the categories, Item 9, “Over the past 7 days the treatment has been cumbersome for me,” was generated (Table 1). A response scale was chosen, where 0 = *not at all* to 4 = *very much*.

**Table 1:** Example patient statements from the development of the QOLPAD

Item #	Item content	Quotation
1	Walking difficulties	“The pain-free walking distance is becoming shorter and shorter; necessary breaks become longer and longer; you cannot walk as you want to anymore.” “Everything is slower because I must take breaks.”
2	Leg pain	“Walking is no longer possible after 10–15 m due to severe pain; there is also pain at rest, but it is even stronger when walking.” “After 100 or 250 m severe pain in the lower legs”
3	Leg paresthesia	“Numbness in the toes; toes feel like they are frozen; a type of tingling that prevents sleeping at night” “It started with tingling, now pain (longer and at shorter intervals) and numbness in the foot; stinging/burning in the calves”
4	Open sores on legs	“Wound on the foot for 8 years, wound hurts especially when walking” “Open sores”
5	Help in everyday life	“Help with grocery shopping and household chores” “Household is no longer manageable, dependent on help, grocery shopping no longer possible”
6	Take part in everyday life	“Less active” “Everything becomes complicated.” “Driving is only possible to a limited extent.”
7	Working life	“No longer able to work in previous jobs; no longer able to walk long distances, have to interrupt (…) longer walking distances at work because of the severe pain” “Contact with customers can no longer be maintained in the same way as it could without the circulatory disorder.”
8	Spare time	“Long-term plans (e.g., holidays) no longer possible for fear of renewed occlusion of the artificial artery” “No sports”
9	Treatment	“Constant visits to the doctor and physiotherapy” “Taking tablets every day; frequent hospital stays”
10	Social environment	“Neglecting the social environment due to listlessness and the physical condition” “Less resilient, therefore less time with other dog owners”
11	Ill-humored	“No normal acting, living, thinking possible; demotivation; life not worth living without help” “At times, feeling of being angry”
12	Worries about illness worsening	“Fear of amputation” “Fear of surgery”

The QOLPAD items refer to physical and mental health, participation in everyday life, work life, leisure activities, and family life. In the cognitive debriefing, only minor changes in wording were suggested by the participants.

### Validation of the QOLPAD

**Fig. 2:**
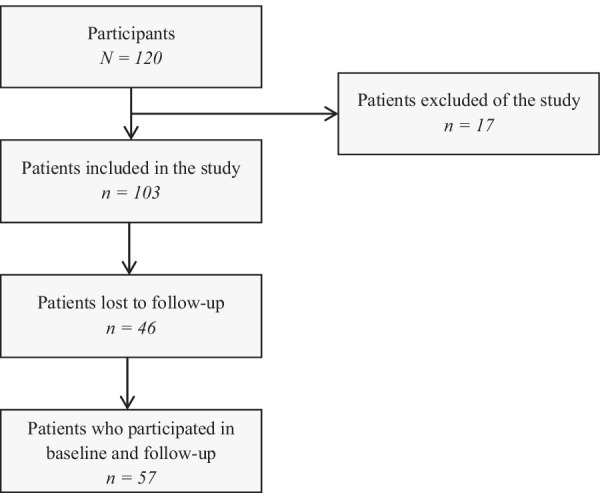
Flow diagram of study population (phase 2, validation study). n: number of patients; baseline: first measurement; follow-up: second measurement, approximately 3 months after the start of treatment

Of the 120 patients included at baseline, 17 were not included in the analysis dataset. These patients had been advised to change their lifestyle but did not receive any medical treatment after completing the baseline assessment; therefore, they did not fulfill the inclusion criteria. Of the 103 patients analyzed at baseline, 57 (55.3%) also participated in the follow-up three months later (Fig. 2). Forty-six patients (44.7%) were lost to follow-up for various reasons, including non-response to phone and mail contact (n = 13), not returning the questionnaire despite reminders (n = 15), organizational challenges (n = 10), refusal of further participation (n = 3), death (n = 3), or planned treatment not being performed due to cancer or rejection (n = 2).

The average age of the study population at baseline was 68.6 ± 10.2 years (range 44–90 years); 70 participants (68.0%) were male and 33 (32.0%) were female (Table 2). The majority of participants were retired (74.2%). The most common PAD stage at baseline was IIb (58.3%). Approximately three months after the start of treatment (follow-up), the most prevalent PAD severity was stage I (41.2%). The most frequently applied treatment was surgical procedure (e.g., bypass surgery and/or thromboendarterectomy) (49.5%), followed by minimally invasive methods (39.8%; Table 2). The participant characteristics were similar in the subgroups of patients who participated at both time points.

**Table 2:** Characteristics of the study population (validation study)

	All patients at baseline		
		n = 103	%
Stage of PAD, n (%)	I	1	1.0
	IIa	9	8.7
	IIb	60	58.3
	III	12	11.7
	IV	18	17.5
	Missing	3	2.9
Sex, n (%)	Male	70	68.0
	Female	33	32.0
Age (years)	Mean ± SD	68.6 ± 10.2	
Treatment	Bypass surgery	12	11.7
	Bypass recanalization	2	1.9
	Bypass and thromboendarterectomy	1	1.0
	Thromboendarterectomy	35	34.0
	Amputation	1	1.0
	Percutaneous transluminal angioplasty	39	37.9
	Urokinase and Percutaneous transluminal angioplasty	1	1.0
	Sympathectomy	1	1.0
	Structured vascular exercise	4	3.9
	Prostaglandin E1	2	1.9
	Missing	5	4.9

Baseline: first measurement; n: number of patients; PAD: peripheral artery disease; SD: standard deviation. PAD stage was classified according to Fontaine, with 1–5 denoting stage I–IV, respectively [3]

### Internal consistency

Cronbach’s alpha was 0.74 at baseline and 0.84 at follow-up. As both values are > 0.7, they indicate sufficient internal consistency of the QOLPAD at both measurement times [12].

### Item responses and missing data

Each item was answered by at least 92 (89.3%) participants at baseline and by at least 44 (89.5%) participants at follow-up (Table 3). Among the patients who completed the QOLPAD at baseline, 78 (76.5%) had no missing items, 17 (16.7%) had one missing item, four (3.9%) had two missing items, and three (3.0%) had between three and six missing items (median, 0; mean, 0.36; SD, 0.85; n = 102). At follow-up, 36 (65.5%) participants had no missing items, 14 (25.5%) had one missing item, three (5.5%) had two missing items, and two (3.6%) had three or four missing items (median, 0; average, 0.49; SD, 0.84; n = 55). The most frequent item not answered was “*I have been affected by the illness in my working life*” at both measurement times (10.7% at baseline and 22.8% at follow-up).

**Table 3:** Scores and missing values of QOLPAD items at baseline and follow-up

		Baseline									Follow-up								
Item		Score (%)					Missing values (%)	Mean	Median	SD	Score (%)					Missing values (%)	Mean	Median	SD
#	Content	0	1	2	3	4					0	1	2	3	4				
1	Walking difficulties	2.0	6.9	14.9	35.6	40.6	1.9	3.1	3.0	1.0	27.8	24.1	20.4	20.4	7.4	5.3	1.6	1.0	1.3
2	Leg pain	4.0	13.0	14.0	39.0	30.0	2.9	2.8	3.0	1.1	35.2	33.3	14.8	14.8	1.9	5.3	1.2	1.0	1.1
3	Leg paresthesia	30.0	19.0	19.0	19.0	13.0	2.9	1.7	2.0	1.4	43.4	18.9	17.0	13.2	7.5	7.0	1.2	1.0	1.3
4	Open sores on legs	87.9	2.0	3.0	2.0	5.1	3.9	0.3	0.0	1.0	87.3	1.8	1.8	3.6	5.5	3.5	0.4	0.0	1.1
5	Help in everyday life	69.0	16.0	5.0	7.0	3.0	2.9	0.6	0.0	1.1	75.5	15.1	5.7	1.9	1.9	7.0	0.4	0.0	0.8
6	Take part in everyday life	12.0	17.0	34.0	15.0	22.0	2.9	2.2	2.0	1.3	18.2	7.3	25.5	25.5	23.6	3.5	2.3	2.0	1.4
7	Working life	70.7	6.5	9.8	4.3	8.7	10.7	0.7	0.0	1.3	79.5	4.5	2.3	2.3	11.4	22.8	0.6	0.0	1.4
8	Spare time	13.1	11.1	23.2	18.2	34.3	3.9	2.5	3.0	1.4	28.3	13.2	32.1	15.1	11.3	7.0	1.7	2.0	1.3
9	Treatment	51.0	15.6	19.8	9.4	4.2	6.8	1.0	0.0	1.2	37.3	21.6	17.6	15.7	7.8	10.5	1.4	1.0	1.3
10	Social environment	4.1	3.1	15.5	11.3	66.0	5.8	3.3	4.0	1.1	5.9	9.8	21.6	17.6	45.1	10.5	2.9	3.0	1.3
11	Ill-humored	30.7	23.8	12.9	18.8	13.9	1.9	1.6	1.0	1.4	38.2	20.0	14.5	14.5	12.7	3.5	1.4	1.0	1.5
12	Worries about illness worsening	13.7	19.6	10.8	26.5	29.4	1.0	2.4	3.0	1.4	27.3	20.0	10.9	21.8	20.0	3.5	1.9	2.0	1.5

Baseline: first measurement; follow-up: second measurement, approximately 3 months after the start of treatment; SD: standard deviation

At baseline, neither floor effects nor ceiling effects were identified with regard to the QOLPAD global score. At follow-up, two patients (3.7%) had the lowest possible score of 0, indicating a small floor effect; there were no ceiling effects at follow-up.

At baseline (i.e., prior to treatment), the highest impairments were found for the item “*I have had difficulties walking*” (76.2% of patients responded with “*quite a lot*” or “*very much*”), followed by “*I have had pain in my legs*” (69.0%). The item with the lowest level of agreement was “*I have had open sores on my legs*” (7.1%), followed by “*I have required help in my everyday life*” (10.0%), and “*I have been affected by the illness in my working life*” (13.0%; Table 3). At follow-up (i.e., approximately three months after the start of treatment), the items that showed the highest change were “*I have had difficulties walking*” (76.2% to 27.8% of patients responded with “*quite a lot/very much*”), and “*I have had pain in my legs*” (from 69.0% to 16.7%). High skewness was found for the items “*I have had open sores on my legs*” (87.9% of patients responded with “*not at all”*), “*I have required help in my everyday life*” (69.0%), and “*I have been affected by the illness in my working life*” (70.7%; Table 3).

### Distribution of the EQ-5D-3L, EQ VAS and the VascuQoL

Prior to treatment (baseline), the EQ-5D-3L score ranged from 0.08 to 1.00, with a median of 0.59. At follow-up, the score ranged from 0.18 to 1.00, with a median of 0.88. The EQ VAS showed median scores of 50.0 at baseline (range: 10.0 to 97.0) and 70.0 at follow-up (range: 30.0 to 100.0). Before treatment, the values for the VascuQoL ranged from 1.6 to 6.6, with a mean of 3.8 (SD = 1.2). The mean value after treatment was 5.1 (SD = 1.5), with a range from 1.8 to 7.0. Thus, all three measures indicated a higher HRQoL after treatment than at the baseline.

### Convergent validity

For convergent validity, we expected stronger correlations with the disease-specific VascuQoL instrument than with the generic EQ-5D-3L and EQ VAS owing to the higher proximity of constructs; this was confirmed by the data (Tables 4, 5). In addition, as expected, we found a significant positive correlation between QOLPAD and both current impairment due to PAD and stage of PAD.

**Table 4:** Correlation of the QOLPAD with convergent criteria at baseline and distribution of convergent criteria

	r	p	n	Mean	Median	Minimum	Maximum
EQ-5D-3L	− 0.62	< 0.001	99	0.57*	0.59	0.08	1.0
EQ VAS	− 0.44	< 0.001	100	55.0*	50.0	10.0	97.0
VascuQoL	− 0.77	< 0.001	101	3.8	3.8	1.6	6.6
Stage of PAD	0.40	< 0.001	98	3.4*	3.0	1	5
Global rating of impairment	0.64	< 0.001	100	2.9*	3.0	0	4

Baseline: first measurement, r: Spearman’s correlation coefficient, p: level of significance, n: number of patients; EQ VAS: EuroQol visual analogue scale, VascuQoL: Vascular Quality of Life Questionnaire, PAD: peripheral artery disease

The stage of PAD was classified according to Fontaine, with 1–5 denoting stage I–IV, respectively [3]

*Variables not normally distributed

**Table 5:** Correlation of the QOLPAD with convergent criteria at follow-up and distribution of convergent criteria

	r	p	n	Mean	Median	Minimum	Maximum
EQ-5D-3L	− 0.81	< 0.001	52	0.78*	0.88	0.18	1.0
EQ VAS	− 0.79	< 0.001	52	66.7*	70.0	30.0	100.0
VascuQoL	− 0.87	< 0.001	54	5.1	5.1	1.8	7.0
Stage of PAD	0.67	< 0.001	49	3.3*	3.0	1	5
Global rating of impairment	0.71	< 0.001	53	1.7*	2.0	0	4

Follow-up: second measurement, approximately 3 months after the start of treatment, r: Spearman’s correlation coefficient, p: level of significance, n: number of patients; EQ VAS: EuroQol visual analogue scale, VascuQoL: Vascular Quality of Life Questionnaire, PAD: peripheral artery disease

The stage of PAD was classified according to Fontaine, with 1–5 denoting stage I–IV, respectively [3]

*Variables not normally distributed

The item intercorrelations of the QOLPAD at the baseline are listed in “Appendix 1”. Item intercorrelations varied widely from − 0.34 to 0.69 (“Appendix 1”).

### Sensitivity to change

In the patients who provided data at both time points, the average QOLPAD global score decreased significantly from 1.5 ± 0.67 before, to 1.2 ± 0.77 after treatment (p = 0.001, n = 52, effect size d = 0.42), indicating responsiveness of the instrument. This was supported by the finding that changes in the QOLPAD were significantly associated with changes in the convergent criteria (generic and disease-specific HRQoL, global rating of impairment, PAD stage) and with the benefit indicators measured at follow-up (patient evaluation of treatment effectiveness, patient recommendation of the treatment, and patient assessment of symptom improvement; Table 6). The QOLPAD scores improved in the two most common treatment groups to a similar extent (minimally invasive treatment: mean change: 0.35; SD, 0.73; p = 0.94; surgical treatment: mean change: 0.34; SD, 0.57; p = 0.94).

**Table 6:** Longitudinal correlations with change in QOLPAD from baseline to follow-up (sensitivity to change)

	r	p	n	Mean	Median	Minimum	Maximum
Δ EQ-5D-3L	− 0.50	< 0.001	51	0.18*	0.12	− 0.36	91.2
Δ EQ VAS	− 0.57	< 0.001	50	10.5	10.0	− 40.0	90.0
Δ VascuQoL	− 0.72	< 0.001	52	1.1	1.0	− 1.2	4.5
Δ Stage of PAD	0.46	< 0.001	46	1.0*	1.0	− 2	4
Δ Global rating of impairment	0.64	< 0.001	50	1.3*	1.0	− 1	4
Global rating of symptom improvement	− 0.55	< 0.001	51	1.7*	1.0	1	4
Global rating of treatment effectiveness	0.46	0.001	51	2.8*	3.0	0	4
Global rating of recommendation of treatment	0.47	0.001	51	3.2*	4.0	0	4

Baseline: first measurement, follow-up: second measurement, approximately 3 months after treatment, r: Spearman’s correlation coefficient, p: level of significance, n: number of patients; EQ VAS: EuroQol visual analogue scale, VascuQoL: Vascular Quality of Life Questionnaire, PAD: peripheral artery disease

The stage of PAD was classified according to Fontaine, with 1–5 denoting stage I–IV, respectively [3]

*Variables not normally distributed

### Feasibility

At baseline, 86.4% of the participants rated the questionnaire as “*very easy*” or “*easy*” to complete, compared to 2.9% who found it “*difficult*.” None of the patients responded “*very difficult*.” Among the respondents who did not participate in the second survey at follow-up (n = 46), only one (2.2%) rated completion as “*difficult*” at baseline.

At follow-up, 85.7% of respondents assessed the QOLPAD as “*very easy*” or “*easy*” to complete; 3.6% chose “*difficult*,” and none chose “*very difficult*.”

## Discussion

This study aimed to develop and validate a short, disease-specific HRQoL instrument for use in patients with PAD. Patients with PAD are known to show rather low adherence, and dropout rates are often higher than those in studies of other patient groups [18]. Therefore, it is particularly important to use easy and short questionnaires, which, nevertheless, are able to determine patient-important burdens of PAD.

The new QOLPAD showed good psychometric properties, including high internal consistency at both measurement time points and convergent validity regarding the HRQoL of generic and disease-specific instruments. Sensitivity to change was supported by significant correlations between change in QOLPAD and changes in convergent criteria; however, the high number of dropouts limits the generalizability of this finding.

Most participants in our study rated the QOLPAD as “*very easy*” or “*easy*” to complete. Although many patients were lost to follow-up, there were few missing data in single items of the questionnaire, with each item being answered by at least 89% of participants at both baseline and follow-up. However, for some items, there was a high number of patients who had low or no impairment, including “*I have had open sores on my legs,” “I have required help in my everyday life*,” and “*I have been affected by the illness in my working life*.” The first item was only relevant for patients with PAD stage IV and was applied to only 18 patients (17.5%) at baseline. Nevertheless, we regard this item as important, as the QOLPAD should be applicable to patients with all stages of PAD, as well as those with high impairment levels with a particularly high need for improvement. The questionnaire can be used in all PAD stages; therefore, it is necessary to assess a variety of symptoms and impairments, which also leads to the inclusion of questions that may not be applicable to all patients in various PAD stages. Therefore, an additional response choice, such as “does not apply,” could be added in a future questionnaire version. However, this needs to be investigated closely to ensure that it is interpreted correctly. The low impairment of working life can be explained by the fact that the majority of participants were retired, and these participants might not have read or followed the QOLPAD instruction that they should tick “*not at all*” in this case. However, the aspect of working life was mentioned in the item collection survey by ten out of 50 patients; therefore, it can be assumed that this item is highly relevant to patients who are still working. It was also found to be an important aspect of HRQoL in patients with PAD in a systematic review by Aber et al. [9]. Therefore, the QOLPAD contains at least one item for each of the six important aspects found in the review. When patients who do not work tick “*not at all*” in the respective item, HRQoL impairment may be underestimated. Again, this may be solved by a “does not apply” choice, or by an additional question on whether the patient is working and excluding the work life item from global score calculation for non-working patients.

The one-page QOLPAD consisted of 12 questions with a five-step response scale. This is considerably shorter than the widely used and recommended seven-page VascuQoL, which contains 25 questions with seven response options that differ between the items. Therefore, the time needed for completion was longer for the VascuQoL than for the QOLPAD. In addition, the VascuQoL, as well as its recently developed short version, the VascuQoL-6 [11], do not cover the aspect of burdens in work life, which is important to non-retired patients. Therefore, the QOLPAD may serve as an alternative to the VascuQoL, especially in younger patient collectives (who have not yet retired) and situations in which brevity is of particular importance, such as in clinical practice or clinical trials where a range of different patient-reported outcomes need to be measured. Nevertheless, due to the short design of the questionnaire with few items, the gathered information is less differentiated and detailed than the data collected with the VascuQoL; by including only one to four items per domain, slight differences in HRQoL between patients as well as over time will be more difficult to detect.

One advantage of the QOLPAD over existing instruments is that it can be used together with the corresponding instrument PBI-PAD, which evaluates treatment goals and therapeutic benefits from the patient’s point of view, while the QOLPAD evaluates current HRQoL impairment. Assessing the QOLPAD both before and after treatment allows detection of the improvement that can be assigned to the treatment, while the PBI-PAD provides a retrospective, direct evaluation of the benefit by the patient. The indirect (pre-post) measurement of change in the HRQoL includes the risk of being affected by response shift bias, while the direct (retrospective) measurement of change may be impaired by recall bias [16]. Hence, it is recommended to combine both measurement approaches in clinical studies, ideally using highly complementary instruments that assess similar content. This can be achieved using both QOLPAD and PBI-PAD.

Given that existing HRQoL instruments have different advantages and disadvantages, a head-to-head comparison could support instrument selection for individual studies [19].

## Limitations

The limitations of this study include the small and narrow study population, including only patients receiving specific interventions, and the high dropout rate. The latter can most likely be attributed to the known low adherence of patients with PAD [18]. An additional reason for non-completion in this study might be that, in the majority of cases, the follow-up questionnaire was sent to participants’ homes instead of asking them to complete it at the clinics, which may have reduced the motivation to complete and return them. However, we found that the patients with and without follow-up data were similar in terms of age, sex, and PAD stage. Due to the small number of patients who participated in the follow-up, the findings on sensitivity to change may be biased and should be interpreted with caution. For example, patients with higher HRQoL improvements may have been more motivated to return the follow-up questionnaire, which would result in the sensitivity findings being less generalizable to the overall patient population. Test-retest reliability should be evaluated in future studies, as well as the psychometric properties in other PAD patient groups. Owing to the limited sample size, no factor analysis was performed. Partly low item intercorrelations need to be examined in future studies to check for unidimensionality. Finally, clinical severity could only be classified using the Fontaine classification, as follow-up was predominantly conducted by mail.

## Conclusion

The QOLPAD is a short and feasible disease-specific instrument that can be used to evaluate HRQoL in patients with PAD in clinical routine or research. It can also be used together with the simultaneously developed questionnaire Patient Benefit Index for PAD (PBI-PAD), as both instruments measure complementary aspects of a patient’s situation, focusing either on HRQoL (QOLPAD) or on treatment needs and treatment benefits (PBI-PAD). This study provided evidence that the QOLPAD is internally consistent and valid in patients in Germany receiving treatment for PAD and provided limited evidence on its sensitivity to change.

## Data Availability

The datasets used and/or analyzed during the current study are available from the corresponding author upon reasonable request.

## Appendix 1

See Table 7.

**Table 7:** QOLPAD item intercorrelations (Spearman’s correlation coefficient) at baseline

Item #	1	2	3	4	5	6	7	8	9	10	11
2	0.69										
3	0.42	0.27									
4	0.06	− 0.03	0.05								
5	0.26	0.13	0.24	0.29							
6	− 0.34	− 0.19	− 0.31	− 0.05	− 0.30						
7	0.17	0.21	0.25	− 0.06	− 0.05	− 0.13					
8	0.13	0.17	< 0.01	0.08	0.24	− 0.12	0.18				
9	0.21	0.15	0.18	0.27	0.30	− 0.15	0.17	0.12			
10	− 0.23	− 0.13	− 0.14	− 0.24	− 0.26	0.27	− 0.10	− 0.11	− 0.24		
11	0.34	0.31	0.36	0.19	0.32	− 0.25	0.21	0.19	0.40	− 0.29	
12	0.34	0.36	0.26	− 0.07	− 0.01	− 0.21	0.22	− 0.02	0.23	− 0.15	0.50

## Abbreviations

EQ VAS: EuroQol visual analogue scale
HRQoL: Health-related quality of life
IVDP: Institute for Health Services Research in Dermatology and Nursing
n: Number of patients
p: Level of significance
PAD: Peripheral artery disease
PBI-PAD: Patient Benefit Index for peripheral arterial disease
QOLPAD: Quality of Life questionnaire for patients with peripheral artery disease
r: Spearman’s correlation coefficient
SD: Standard deviation
SF-36: 36-Item Short Form Health Survey
UKE: University Medical Center Hamburg-Eppendorf
VascuQoL: Vascular Quality of Life questionnaire
VascuQoL-6: Six-item version of Vascular Quality of Life questionnaire
